# A novel approach to preventing adverse events after circumferential endoscopic submucosal dissection of the ileocecal valve.

**DOI:** 10.1055/a-2882-8286

**Published:** 2026-06-17

**Authors:** Davide Massimi, Matteo Spertino, Antonio Capogreco, Ludovico Alfarone, Cesare Hassan, Roberta Maselli, Alessandro Repici

**Affiliations:** 1 Endoscopic Unit, Department of Gastroenterology 9268 IRCCS Humanitas Research HospitalRozzanoMilanItaly; 2 Department of Biomedical Sciences 437807 Humanitas UniversityPieve EmanueleItaly

Endoscopic submucosal dissection (ESD) involving the ileocecal valve (ICV) remains a technically challenging procedure due to the anatomical complexity, limited maneuverability, and the thin submucosal layer in this region. 1​ In certain cases, particularly those involving large laterally spreading tumors (LSTs), circumferential resection may be required; however, this approach is associated with a significant risk of adverse events. 2​ ​3​

A 71-year-old patient was referred for a recurrent 30-mm homogeneous granular LST (Kudo pit pattern IIIL), after three prior incomplete endoscopic resections, involving both lips of the valve circumferentially and extending 1 cm into the terminal ileum ( Video 1 ).

**Video 1** A dual approach to prevent adverse events after circumferential ICV endoscopic submucosal dissection using selective cecal clipping and application of a peptide matrix to the colonic margin to promote controlled mucosal remodeling.


**Fig. 1:**
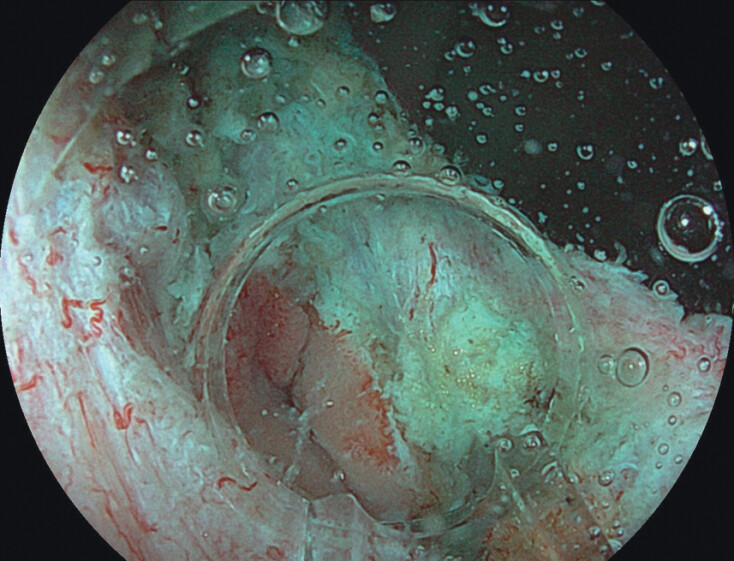
Circumferential defect after complete dissection.

The procedure began with a circumferential incision along the ileal margin, followed by an incision on the colonic side. The lesion was resected using an underwater ESD technique with a multitunneling approach, 4​ performed with a colonoscope fitted with a conic hood ( Fig. 1 ).

An integrated needle-tipped IT knife was used with Swift Coag (Effect 4.0) for coagulation and selected phases of dissection, and Endo Cut Q (Effect 3-3) for mucosal incision and dissection.

**Fig. 2:**
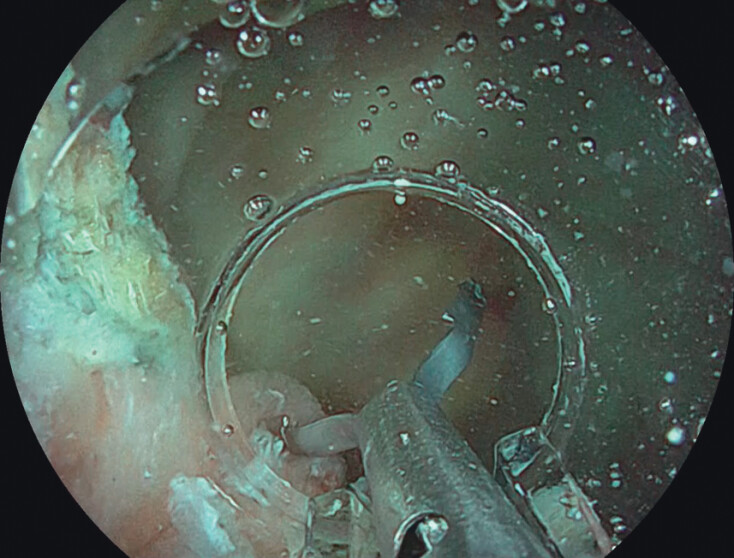
Use of an endoscopic clip to evert the ileal margin and fix it to the distal colonic margin.

To prevent post-ESD complications, a dual strategy was employed: (1) the ileal margin was everted and fixed to the distal colonic margin using endoscopic clips, achieving partial closure over approximately 180° of the defect ( Fig. 2 ); (2) on the contralateral side, where clip placement on the proximal colonic margin was technically challenging, a self-assembling peptide matrix was applied. This approach aimed to avoid a circumferential mucosal defect, thereby promoting asymmetric healing and supporting granulation and re-epithelialization of the unclipped colonic margin. No adverse events occurred.

Histology confirmed an R0 resection. At 3-month and 1-year follow-ups, the ICV appeared widely patent.

This video illustrates a reproducible strategy to prevent stenosis after circumferential ICV ESD. Although this peptide matrix is primarily used for hemostasis, preclinical studies suggest a potential role in reducing fibrosis. 5​

Endoscopy_UCTN_Code_TTT_1AQ_2AD_3A
